# Motor, Visual and Emotional Deficits in Mice after Closed-Head Mild Traumatic Brain Injury Are Alleviated by the Novel CB2 Inverse Agonist SMM-189

**DOI:** 10.3390/ijms16010758

**Published:** 2014-12-31

**Authors:** Anton Reiner, Scott A. Heldt, Chaela S. Presley, Natalie H. Guley, Andrea J. Elberger, Yunping Deng, Lauren D’Surney, Joshua T. Rogers, Jessica Ferrell, Wei Bu, Nobel Del Mar, Marcia G. Honig, Steven N. Gurley, Bob M. Moore

**Affiliations:** 1 Department of Anatomy and Neurobiology, the University of Tennessee Health Science Center, Memphis, TN 38163, USA; E-Mails: sheldt@uthsc.edu (S.A.H.); nhart2@uthsc.edu (N.H.G.); aelberge@uthsc.edu (A.J.E.); ydeng@uthsc.edu (Y.D.); ldsurney@uthsc.edu (L.D.); jroger45@uthsc.edu (J.T.R.); jferre13@uthsc.edu (J.F.); wbu1@uthsc.edu (W.B.); ndelmar@uthsc.edu (N.D.M.); mhonig@uthsc.edu (M.G.H.); 2 Department of Ophthalmology, the University of Tennessee Health Science Center, Memphis, TN 38163, USA; 3 Department of Pharmaceutical Sciences, the University of Tennessee Health Science Center, Memphis, TN 38163, USA; E-Mails: cpresle4@uthsc.edu (C.S.P.); sngurley@comcast.net (S.N.G.); bmoore@uthsc.edu (B.M.M.)

**Keywords:** TBI, deficits, microglia, CB2 receptors, therapy

## Abstract

We have developed a focal blast model of closed-head mild traumatic brain injury (TBI) in mice. As true for individuals that have experienced mild TBI, mice subjected to 50–60 psi blast show motor, visual and emotional deficits, diffuse axonal injury and microglial activation, but no overt neuron loss. Because microglial activation can worsen brain damage after a concussive event and because microglia can be modulated by their cannabinoid type 2 receptors (CB2), we evaluated the effectiveness of the novel CB2 receptor inverse agonist SMM-189 in altering microglial activation and mitigating deficits after mild TBI. *In vitro* analysis indicated that SMM-189 converted human microglia from the pro-inflammatory M1 phenotype to the pro-healing M2 phenotype. Studies in mice showed that daily administration of SMM-189 for two weeks beginning shortly after blast greatly reduced the motor, visual, and emotional deficits otherwise evident after 50–60 psi blasts, and prevented brain injury that may contribute to these deficits. Our results suggest that treatment with the CB2 inverse agonist SMM-189 after a mild TBI event can reduce its adverse consequences by beneficially modulating microglial activation. These findings recommend further evaluation of CB2 inverse agonists as a novel therapeutic approach for treating mild TBI.

## 1. Introduction

Mild traumatic brain injury (TBI) is a common occurrence during military combat, sports, recreational activities, and vehicle use [1,2]. Mild TBI, which involves either brief or no loss of consciousness and causes minimal overt brain destruction, typically produces widespread axonal injury that is commonly referred to as “diffuse” axonal injury [3,4]. Mild TBI leads to a variety of adverse sensory, motor, cognitive and emotional outcomes [1,2,3]. The initial injury with mild TBI appears to stem from the brain tissue deformation that results from the shock wave transmitted through brain and CSF by the blast impact, or from the brain compression—expansion during rapid head acceleration—deceleration [5]. The concussive insult leads to axonal injury and cell–cell signaling defects, and can set in motion subsequent secondary degenerative events. Although many approaches have been tested in animal models and human clinical trials [6], effective therapies for mitigating the effects of mild TBI have not been developed. Since microglial activation is thought to play a role in the adverse outcome after mild TBI [7,8,9,10,11,12,13,14], we used our focal blast model of mild TBI in mice [15,16] to evaluate the benefits of a novel pharmacological approach for reducing the adverse actions of activated microglia after TBI.

Activated microglia can exist in either of two states, an M1-state associated with production of pro-inflammatory cytokines and reactive oxygen species and an anti-inflammatory M2-state associated with wound healing and debris clearance [17]. Because of the role that M1-state activated microglia appear to play in furthering the injury process after a concussive event [7,8,9,10,11,12,13,14], we focused on modulation of microglial activation state via their cannabinoid type 2 receptors (CB2) as a therapeutic approach [18]. CB2 receptors, in contrast to cannabinoid type 1 (CB1) receptors, are non-psychotropic, in large part because they are primarily localized to microglia rather than neurons in brain [19,20,21]. Activated microglia rapidly increase their expression of CB2 receptors [18,19,20,21,22], and so drugs acting on CB2 are especially promising for selectively targeting microglia for therapeutic purposes after brain injury. Moreover, CB2 drugs have limited side effects compared to other anti-inflammatory agents. Agonist binding to CB2 receptors (which are negatively coupled to adenylyl cyclase) attenuates microglial M1 activation and cytokine release [20,23]. Although CB2 agonists have shown benefit in some neurodegenerative diseases in which microglial activation plays a role [24,25,26,27], they have not shown consistently strong benefit in treating TBI [28,29,30,31,32]. CB2 antagonists would be inadvisable as an alternative, since they exacerbate M1 microglial activation [25,33,34].

CB2 inverse agonists represent a unique class of ligands with promise for beneficially modulating microglia to treat TBI [35,36]. CB2 inverse agonists lock constitutively active CB2 receptors into an inactive state and thus produce the opposite action of a CB2 agonist—they reduce adenylyl cyclase inhibition and thereby increase cyclic adenosine monophosphate (cAMP) production [37]. This in turn leads to downstream activation of protein kinase A (PKA), which phosphorylates the cAMP response element binding protein (CREB). Increased phosphorylation and nuclear translocation of CREB appear to underlie both an anti-inflammatory effect and a pro-repair effect of CB2 inverse agonists, via a phenotype shift in activated microglia from the M1 to the M2 state [35,36,38]. We therefore tested if a CB2 inverse agonist could improve the outcome in mice using our focal air pressure blast model to create mild TBI [15,16]. We used the selective CB2 inverse agonist SMM-189 (compound 5 in reference [39]), developed in our laboratories. We have found that SMM-189 exhibits functional activity only at the CB2 receptor, and others have shown that this structural class of compounds shows good blood-brain barrier penetration [40]. As described below, SMM-189 has striking efficacy in reducing the M1 features and increasing the M2 features of human microglia *in vitro*. Further, we found it reverses motor, sensory, emotional and morphological deficits caused by mild TBI in a mouse focal cranial blast model. Our findings recommend further evaluation of CB2 inverse agonists as a novel therapeutic approach for treating mild TBI.

## 2. Results

### 2.1. Cultured Human Microglia

#### 2.1.1. Cytokines

**Figure 1:**
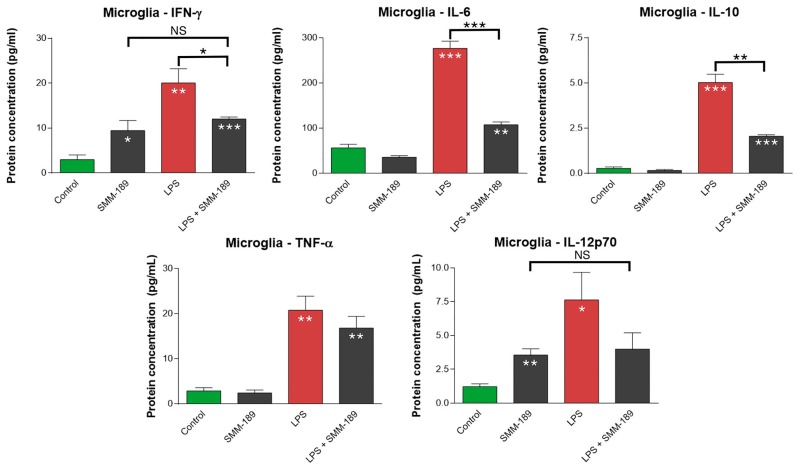
Effect of SMM-189 on cytokines secreted from primary human microglial cells *in vitro*. Primary human microglia were treated with lipopolysaccharide (LPS) or vehicle, and then SMM-189 or vehicle was added one hour later. Cytokines were then assayed 17 h subsequently. LPS significantly increased cytokine levels above that in control. SMM-189 by itself also significantly elevated interferon-gamma (IFN-γ) and interleukin-12p70 (IL-12p70), but to a lesser extent than LPS. SMM-189 attenuated the effect of LPS on cytokine secretion, significantly reducing IFN-γ, interleukin-6 (IL-6) and interleukin-10 (IL-10) below LPS-only levels and restoring IFN-γ and IL-12p70 to SMM-189-alone levels. SMM-189 also trended toward reducing TNFα. Asterisks above bars spanning the LPS-alone and LPS + SMM-189 columns, or the SMM-189-alone and LPS + SMM-189 columns indicate significance levels for the comparison between these two conditions. Asterisks on a column indicate a significant difference from the control condition. NS—Not significant, * *p* < 0.05, ** *p* < 0.005, *** *p* < 0.0005.

To evaluate SMM-189 effects, we started by comparing the levels of cytokines released by primary human microglia cultured under control conditions and those activated with lipopolysaccharide (LPS) in the presence and absence of SMM-189. LPS-treatment significantly increased the levels of interferon-gamma (IFN-γ), interleukin-6 (IL-6), interleukin-10 (IL-10), interleukin-12p70 (IL-12p70), and tumor necrosis factor-α (TNF-α) (*p* < 0.002) (Figure 1), but did not increase the levels of granulocyte-macrophage colony-stimulating factor (GM-CSF), interleukin-2 (IL-2) or interleukin-1β (IL-1β). Addition of SMM-189 reduced the expression of IFN-γ, IL-6, IL-10, and IL-12p70 that occurred in LPS-activated human microglia, so that they were either significantly less (*p* < 0.015) than in the LPS-alone condition (IFN-γ, IL-6, and IL-10), and/or not significantly different than in the control + SMM-189 condition (IFN-γ and IL-12p70) (Figure 1). Thus, four of the five cytokines whose expression by microglia was increased with LPS treatment showed significantly reduced expression when SMM-189 treatment was combined with LPS-treatment, and the remaining cytokine (TNF-α) trended in that direction. By itself, SMM-189 slightly elevated IFN-γ and IL-12p70, but much less so than did LPS alone.

#### 2.1.2. Chemokines

**Figure 2:**
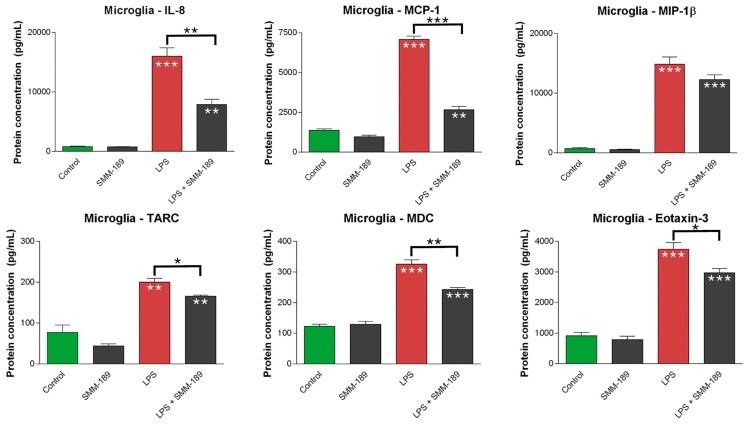
Effect of SMM-189 on chemokines secreted from primary human microglial cells *in vitro*. Primary human microglia were treated with LPS or vehicle, and then SMM-189 or vehicle was added one hour later. Chemokines were then assayed 17 h subsequently. LPS significantly increased chemokine levels above that in control for all chemokines shown. SMM-189 treatment alone had no significant effect on chemokine secretion, other than a slight reducing effect on MCP-1 (monocyte chemoattractant protein-1). SMM-189 significantly reduced the LPS-induced increase in the levels of IL-8 (interleukin-8), MCP-1, TARC (thymus and activation-regulated chemokine, also called chemokine ligand 17, or CCL17), MDC (macrophage-derived chemokine, also called C–C motif chemokine 22, or CCL22), and eotaxin-3 (also called C–C motif chemokine 11, or CCL11), and trended toward doing so for MIP-1β (macrophage inflammatory protein-1-beta, also called chemokine C–C motif ligand 3, or CCL3). Asterisks above bars spanning the LPS-alone and LPS + SMM-189 columns, or the SMM-189-alone and LPS + SMM-189 columns indicate significance levels for the comparison between these two conditions. Asterisks on a column indicate a significant difference from the control condition. * *p* < 0.05, ** *p* < 0.005, *** *p* < 0.0005.

LPS treatment also significantly increased the levels of eight of the nine pro-inflammatory chemokines we tested: IL-8 (interleukin-8), MCP-1 (monocyte chemoattractant protein-1), MIP-1β (macrophage inflammatory protein-1-beta, also called chemokine C–C motif ligand 3, or CCL3), TARC (thymus and activation-regulated chemokine, also called chemokine ligand 17, or CCL17), MDC (macrophage-derived chemokine, also called C–C motif chemokine 22, or CCL22), eotaxin-3, eotaxin-1 (also called C–C motif chemokine 11, or CCL11), and IP-10 (interferon gamma-induced protein-10, also called C–X–C motif chemokine 10 or CXCL10) (Figure 2). LPS treatment did not significantly increase monocyte chemotactic protein-4 (MCP-4). Addition of SMM-189 significantly reduced the LPS-induced elevation in IL-8 (*p* = 0.000053), MCP-1 (*p* = 1.1331 × 10^−10^), MIP-1β (*p* = 0.0308), TARC (*p* = 0.0409), MDC (*p* = 0.000001), and eotaxin-3 (*p* = 4.3072 × 10^−7^). No effect of SMM-189 treatment on the LPS-induced elevation was seen for eotaxin-1 or IP-10. Thus, six of the eight chemokines with increased expression after LPS treatment, showed reduced expression when SMM-189 treatment was combined with LPS-treatment. By itself, SMM-189 did not affect expression of any of the chemokines examined, except for a slight depressing effect on TARC (*p* = 0.04744).

#### 2.1.3. M1 Markers

**Figure 3:**
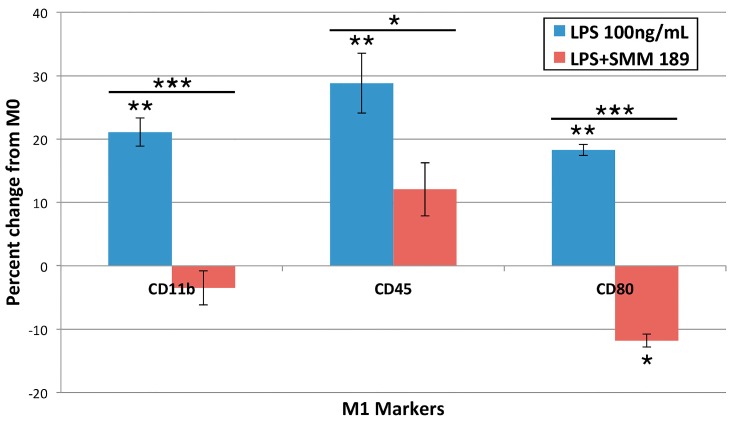
Cell surface receptor expression of M1 microglia phenotype-associated markers CD11b, CD45, and CD80 24 h after treatment with LPS, or LPS + SMM-189. Levels are expressed as percent change from control (M0). LPS significantly increased expression of CD11b, CD45, and CD80, and SMM-189 significantly reversed expression of M1 markers induced by LPS. Asterisks above the LPS-alone or below the LPS + SMM-189 columns indicate significant differences from the M0 condition. Asterisks above bars spanning the LPS-alone and LPS + SMM-189 columns indicate significance levels for the comparison between these two conditions. * *p* < 0.05, ** *p* < 0.005, *** *p* < 0.0005.

We also examined how LPS and SMM-189 affected the expression of several M1 microglial markers, in this case using cultured immortalized human microglia. LPS alone increased the expression of the M1 microglial markers CD11b (*p* = 0.0012), CD45 (*p* = 0.0035), and CD80 (*p* = 0.0016) (Figure 3). SMM-189 co-treatment significantly reduced levels of CD11b (*p* = 0.000498) and CD45 (*p* = 0.04882) below those in the LPS-alone treated groups, so that they were statistically indistinguishable from the untreated condition. SMM-189 treatment decreased CD80 expression to an even greater extent, as it decreased it to less than in both LPS-alone (*p* = 0.012522) and the untreated conditions (*p* = 0.000107).

#### 2.1.4. M2 Markers

**Figure 4:**
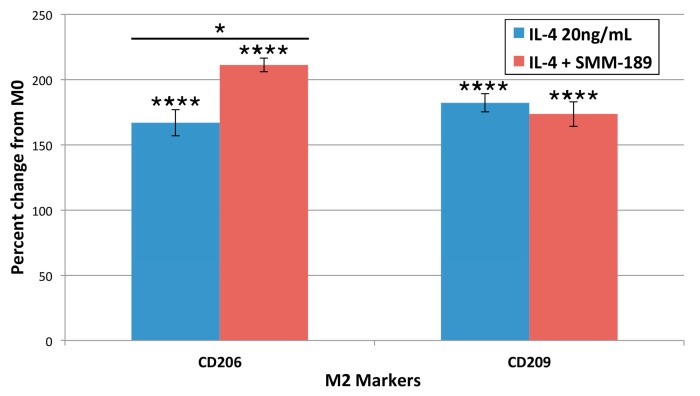
Cell surface receptor expression of the M2 phenotype-associated markers CD206 and CD209 24 h after treatment with interleukin-4 (IL-4), or IL-4 + SMM-189. Note that IL-4 significantly increased expression of both CD206 and CD209, and that SMM-189 significantly increased expression of CD206 beyond that seen with IL-4 alone. Asterisks above bars spanning the LPS-alone and LPS + SMM-189 columns indicate significance levels for the comparison between these two conditions. Asterisks above a column indicate a significant difference from the control condition. * *p* < 0.05, **** *p* < 0.0005.

We next examined the effects of the M2-polarizing stimulant, interleukin-4 (IL-4), on the immortalized human microglia. Treatment with IL-4 led to significant increases in the levels of the M2 markers CD206 (*p* = 0.000006) and CD209 (*p* = 0.000002) on immortalized human microglia (Figure 4). Combined treatment with SMM-189 and IL-4 yielded a statistically significant increase in CD206 above that seen with IL-4 alone (*p* = 0.00734), while no significant change from IL-4 alone was seen in CD209 expression with SMM-189 co-treatment.

#### 2.1.5. Cannabinoid Type 2 Receptors (CB2)

Finally, we examined the effects of a series of M1 or M2 stimulants on CB2 expression by the immortalized human microglia. CB2 levels were significantly increased by all four of the M1-polarizing stimulants we tested: IFN-γ (by 25%), IL-1β (by 150%), LPS (by 70%), and IL-10 (by 35%). The M2 stimulant IL-4 significantly increased CB2 levels on the immortalized microglia by 400%.

### 2.2. Mice with Mild Traumatic Brain Injury (TBI)

#### 2.2.1. Microglial Responses to TBI and SMM-189

**Figure 5:**
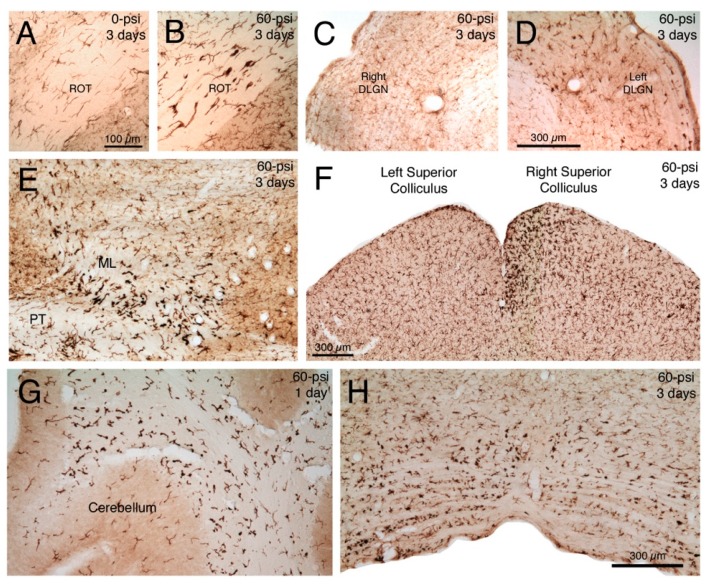
Transverse sections immunostained for ionized calcium-binding adapter molecule-1 (IBA1), showing: Normal microglia in right optic tract (ROT) three days after a left cranial 0-psi blast (**A**); activated microglia in the ROT three days after a left cranial 60-psi blast (**B**); normal microglia in left dorsal lateral geniculate nucleus (DLGN) three days after a left cranial 60-psi blast (**C**); activated microglia in the right DLGN three days after the same left cranial 60-psi blast (**D**); activated microglia in the left medial lemniscus (ML) and pyramidal tract (PT) at the level of the mesencephalon three days after left cranial 60-psi blast (**E**); normal microglia in left superior colliculus and activated microglia (especially medially in the upper layers) in the right superior colliculus three days after a left cranial 60-psi blast (**F**); activated microglia in the deep cerebellar white matter one day after left cranial 60-psi blast (**G**); and activated microglia in basal medulla three days after a left cranial 60-psi blast, particularly on the left side (**H**). Note that normal microglia (**A**,**C**) immunolabel lightly for IBA1, and have a small cell body and thin processes. By contrast activated microglia immunolabel intensely for IBA1, and have more prominent perikarya that possess thick short processes. Images **A** and **B** are at the same magnification as one another, images **C** and **D** are at the same magnification as one another, and images **E**, **G** and **H** are at the same magnification as one another.

**Figure 6:**
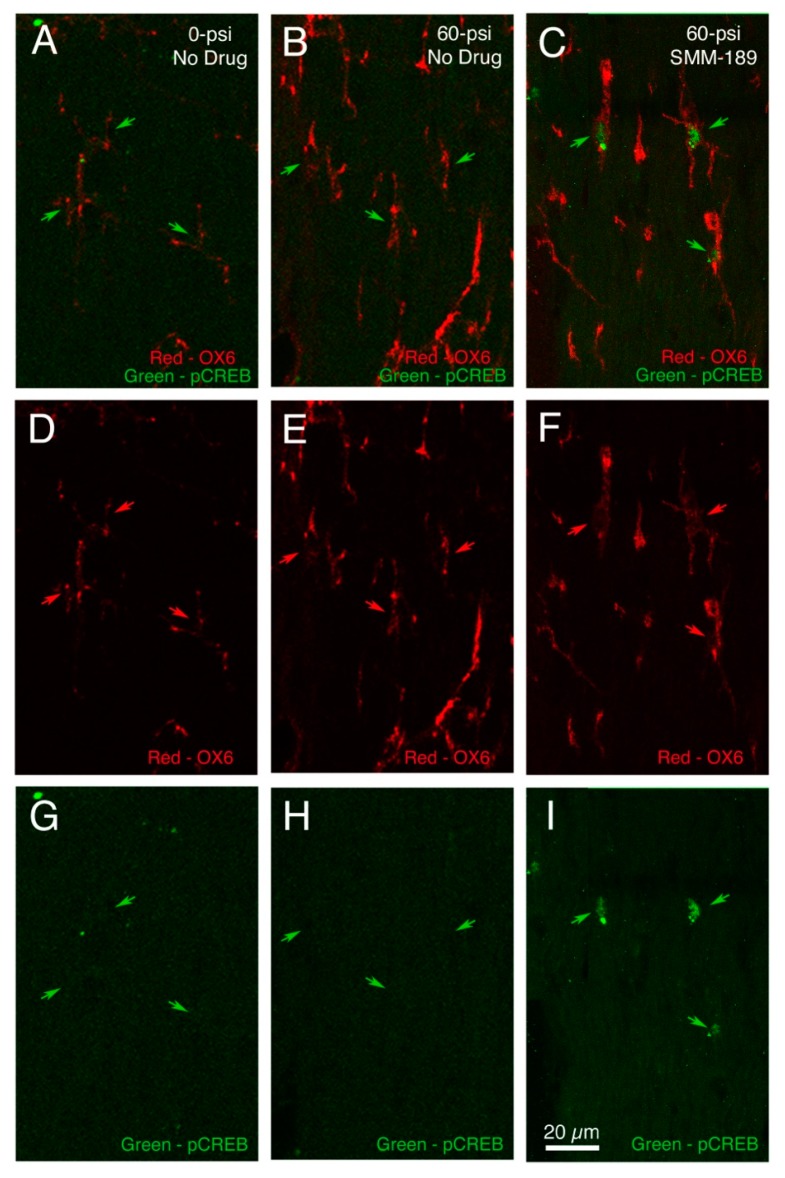
Double immunostaining for the activated microglial marker OX6 (major histocompatibility complex (MHC) class II antigen, red) and phosphorylated-CREB (the cyclic adenosine monophosphate (cAMP) response element binding protein, green) in the right optic tract of a 60-psi blasted mouse treated with SMM-189 (**C**,**F**,**I**) three days after blast compared to a sham blasted mouse not treated with SMM-189 (**A**,**D**,**G**) seven days after sham, and a 60-psi mouse not treated with SMM-189 (**B**,**E**,**H**) five days after blast. The first row (**A**–**C**) shows merged images, while the second (**D**–**F**) and third (**G**–**I**) rows show the red and green channels, respectively. Note that OX-6 immunostaining is more intense and found in more microglia after 60-psi blast (red arrows) compared to sham blast regardless of SMM-189 treatment (second and third columns). Phosphorylated-CREB, however, is evident in microglial nuclei (green arrows) only after SMM-189 treatment (last column).

We have found that axonal pathology is evident in the left optic nerve and right optic tract shortly after left cranial 50–60 psi blasts [16] and is accompanied by microglial activation, as revealed by the change in microglial morphology (shorter processes, larger cell bodies, rod-like appearance), and increased intensity of ionized calcium-binding adapter molecule-1 (IBA1) immunolabeling (Figure 5A,B). The activation was also seen in right optic tract target areas, such as the right dorsal lateral geniculate nucleus (Figure 5C,D) and the upper layers of the right superior colliculus (Figure 5F). In the case of the right optic tract and its target areas, the microglial activation was evident by the third day after blast, more prominent at one week, and no longer prominent by two to eight weeks. We also observed microglial activation in other brain regions where we detected axonal pathology, for example, in the medial lemniscus (Figure 5E), lateral lemniscus, cerebellar peduncles and deep cerebellar white matter (Figure 5G), and pyramidal tract (Figure 5H). Heightened immunolabeling with OX6 confirms microglia are activated after 50–60 psi blast (Figure 6A,B,D,E). Axonal pathology in the right optic nerve was, by contrast, minimal, although some axonal pathology was seen in the left optic tract (possibly representing injured uncrossed left optic nerve axons).

We also examined the effects of SMM-189 treatment on the regulation of activated microglia, in particular in terms of increased CREB signaling, as reflected in increased nuclear levels of phosphorylated-CREB (pCREB). We focused on the optic tract three to seven days after TBI, a site and time of consistent axonal injury as evidenced by the presence of swollen axonal bulbs, which reflect sites of microtubule breakage [41,42]. We found that microglia in the right optic tract (which is the continuation of the left optic nerve) of mice with 60-psi left side cranial blasts are OX6-rich (*i.e.*, major histocompatibility complex (MHC) class II antigen-rich) and enlarged, indicating they are activated (Figure 6B,E). Notably, nearly all activated microglia appear to have prominent nuclear labeling for pCREB after daily SMM-189 commencing two hours after blast (Figure 6C,F). By contrast, microglia are small and not as prominently OX6 + three–seven days after sham blast, and nuclear pCREB is also not prominent Figure 6A). Although OX6 + microglia are numerous and well-labeled after 60-psi blast, without SMM-189 treatment nuclear pCREB is absent (Figure 6B). Thus, SMM-189 does appear to have the expected inverse agonist action on microglia *in vivo*, namely a boost in cAMP-mediated signaling, and an attendant increase in nuclear pCREB.

#### 2.2.2. Motor Function

**Figure 7:**
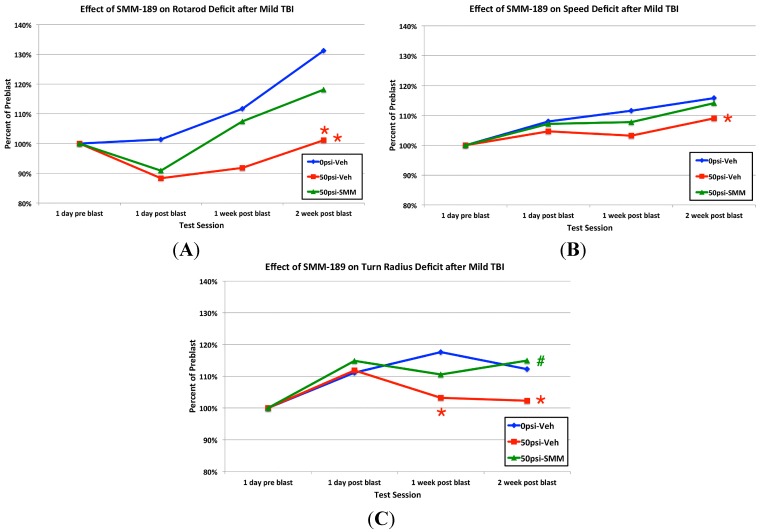
Comparison of the effects of 0-psi blast in vehicle-treated mice, 50-psi blast in vehicle-treated mice, and 50-psi blast in SMM-189-treated mice on rotarod motor performance (**A**); maximum speed in open field (**B**); and turn radius in open field (**C**). Note that motor performance is impaired for vehicle-treated 50-psi mice on all three tests, and restored by SMM-189. Red asterisks for a given time point indicate a significant difference between vehicle-treated 50-psi mice and vehicle-treated 0-psi mice for that time point, while a red asterisk to the right indicates a significant overall difference between vehicle-treated 50-psi mice and vehicle-treated 0-psi mice. At no time point did SMM-189-treated 50-psi mice differ from vehicle-treated 0-psi mice, nor did they show an overall difference. A green **#** indicates a significant difference between SMM-189 and vehicle-treated 50-psi mice.

We used our blast model to characterize the motor deficits produced by 50-psi blasts in three-month old male C57BL/6 mice, and their alleviation by SMM-189 treatment for the two weeks commencing just after blast. A significant overall rotarod deficit across test sessions was observed by ANOVA in 50-psi blast mice receiving vehicle (Figure 7), compared to 0-psi mice receiving vehicle (*p* = 0.013), but not in SMM-189-treated 50-psi mice (*p* = 0.366). Deficits were also seen after 50-psi blast in maximum speed and turn radius in open field (Figure 7). For example, vehicle-treated sham-blasted mice showed significant improvement in maximum speed compared to pre-blast by the first week post blast (*p* = 0.0056), but the vehicle-treated 50-psi mice did not (*p* = 0.5279). By contrast, SMM-189-treated 50-psi mice (Figure 7) did show significant improvement in maximum speed compared to pre-blast by the first week after blast (*p* = 0.0355). Moreover, a significant overall deficit in maximum speed was observed across test sessions by ANOVA in 50-psi blast mice receiving vehicle (Figure 7), compared to 0-psi mice receiving vehicle (*p* = 0.046), but not in SMM-189-treated 50-psi mice (*p* = 0.454). Similarly, post blast mean turn radius was significantly less in vehicle-treated 50-psi mice than in vehicle-treated 0-psi mice (*p* = 0.028) across test sessions by ANOVA, but SMM-189-treated 50-psi mice did not differ from sham mice (*p* = 0.944), but did differ significantly from vehicle-treated 50-psi mice (*p* = 0.038).

**Figure 8:**
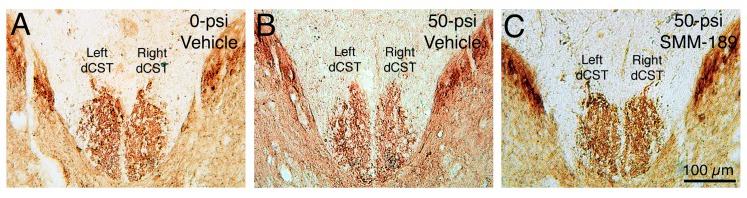
Transverse sections of spinal cord at mid-thoracic levels immunostained for PKC-γ to visualize the dorsal component of the corticospinal tract (dCST). The dCST on the right side of the spinal cord is slightly decreased in fiber enrichment after 50-psi blast in vehicle-treated mice (**B**), compared to vehicle-treated mice with 0-psi blast (**A**). As the axons of pyramidal cortical neurons cross the midline at the spinomedullary junction before descending in the CST, these results are consistent with left pyramidal tract injury by blast to the left side of the cranium. Images are at the same magnification. The right dCST loss is prevented by SMM-189 (**C**).

To investigate the possible basis of the motor deficits and their alleviation by SMM-189, we characterized the corticospinal tract injury in the vehicle-treated 50-psi blast mice, compared to vehicle-treated sham mice and SMM-189-treated 50-psi mice. Although cortical injury itself was not apparent [15], we found a significant atrophy of the right dorsal corticospinal tract (which arises from the left cerebral cortex,* i.e.*, the blasted side) after 50-psi left cranial blast in vehicle-treated mice (Figure 8). For example, the right dorsal corticospinal tract area was only 89.9% of the left, while the right dorsal corticospinal tract area in vehicle-treated sham-blasted mice was 100.4% of left. The difference between vehicle-treated 50-psi blast and vehicle-treated sham-blasted mice was statistically significant (*p* = 0.0058). By contrast, the right dorsal corticospinal tract in SMM-189-treated 50-psi mice was 101.3% of left, and this ratio was not significantly different than in vehicle-treated sham-blasted mice (*p* = 0.6581), but significantly greater than in the vehicle-treated 50-psi mice (*p* = 0.0055).

#### 2.2.3. Visual Function

**Figure 9:**
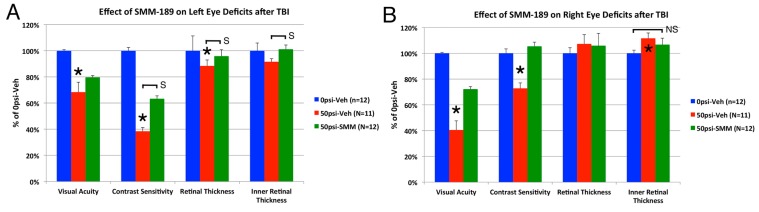
Effect of blast on visual acuity and contrast sensitivity four weeks after blast and retinal thickness 11 weeks after blast in the left eye (**A**) and right eye (**B**) of vehicle-treated sham mice, vehicle-treated 50-psi mice, and SMM-189 treated 50-psi mice. Optomotor testing in vehicle-treated mice with 50-psi blast revealed reduced visual acuity and contrast sensitivity in the left eye and retinal thinning (**A**), compared to vehicle-treated sham mice, which was prevented by SMM-189. Optomotor visual testing in vehicle-treated mice with 50-psi blast also revealed reduced visual acuity and contrast sensitivity in the right eye and retinal thickening, compared to vehicle-treated sham mice (**B**), which were attenuated by SMM-189. Asterisks indicate significant differences between vehicle-treated 50-psi and sham mice, and bars indicate significance (S) or non-significant differences (NS) between the groups spanned by the bar. * *p* < 0.05.

We assessed visual acuity at high contrast (100% contrast) and contrast sensitivity at low spatial frequency (0.042 cycles per degree,* i.e.*, wide stripes) four weeks after blast. Retinal layer thickness was assessed histologically 11 weeks after blast (Figure 9). Visual acuity for both eyes in our vehicle-treated sham blasted mice was indistinguishable from that in normal mice (about 0.4 cycles per degree). Acuity in the left eye of vehicle-treated 50-psi blasts was significantly reduced to 68.3% of vehicle-treated sham left eyes (*p* = 0.000013), and contrast sensitivity was 38.3% of that in vehicle-treated sham left eyes (*p* = 0.000002). The left retina was significantly (11.6%) thinner 11 weeks after 50-psi blast than in sham blasted mice (*p* = 0.026). The most prominent thinning was in the inner nuclear layer (14.2%) and the photoreceptor layers of the outer retina (14.4%), which both were significantly less than in vehicle-treated sham left eyes (*p* = 0.024; *p* = 0.010, respectively). This retinal thinning is likely to have contributed to the left eye visual deficits. SMM-189 treatment reduced the left eye deficit in visual acuity caused by 50-psi blast to 79.7% of sham (Figure 9), trending toward significantly better than in vehicle-treated 50-psi mice (*p* = 0.079). SMM-189 treatment also reduced the left eye deficit in contrast sensitivity to 63.3% of sham, resulting in significantly greater contrast sensitivity than in vehicle-treated 50-psi mice (*p* = 0.00002). Associated with the benefits of SMM-189 for visual function, SMM-189 also reversed the retinal thinning. Overall retinal thickness was no longer significantly less than in vehicle-treated sham-blasted mice (*p* = 0.411). Moreover, inner retinal thickness in left eyes from SMM-189-treated 50-psi mice (101.1% of sham left eyes) was significantly greater than in vehicle-treated 50-psi mice (*p* = 0.0216), and SMM-189 treatment rendered both the inner nuclear layer (*p* = 0.917) and outer retinal thickness (*p* = 0.105) indistinguishable from that in vehicle-treated sham blast mice. In addition, left eye inner nuclear layer thickness in SMM-189 treated 50-psi mice was significantly greater than in vehicle-treated 50-psi mice (*p* = 0.021). Note that the cornea appeared normal and no lens opacities that might have affected visual performance were observed in any of the mice subjected to 50-psi blasts. This was true as well for the right eye, which exhibited visual defects described in the following paragraph.

Right eye acuity in vehicle-treated mice with 50-psi blast to the left side of the cranium (Figure 9) was 40.5% of that in right eyes from vehicle-treated sham-blast mice (*p* = 0.00189 × 10^−8^), and contrast sensitivity was 72.8% of that in sham (*p* = 0.007). Unlike the left eye, a significant 11.6% thickening of the inner retina (*p* = 0.045) was observed in right eyes after 50-psi blast in vehicle-treated mice, resulting in a slightly (7.3%) thicker right retina overall. SMM-189 treatment reversed the visual deficits and appeared to attenuate the retinal thickening. The visual acuity in right eyes from SMM-189 treated mice with 50-psi blast was improved to 72.2% of sham and was significantly greater than in the vehicle-treated 50-psi right eyes (*p* = 0.000008). Similarly, contrast sensitivity in right eyes from SMM-189 treated mice with left-side cranial 50-psi blast was normalized (105.4% of sham) and was significantly greater than for vehicle-treated 50-psi right eyes (*p* = 0.003). SMM-189 appeared to slightly reduce the inner retinal thickening associated with 50-psi blast, since inner retinal thickness was lessened by 42.3% and not significantly different than in sham right eyes (*p* = 0.237). Such attenuation of retinal thickening could have contributed to the rescue of right eye visual acuity and contrast sensitivity deficits observed after left side 50-psi cranial blast. Since inner retinal thickening was, however, not significantly less than in the vehicle-treated 50-psi retinas (*p* = 0.396), further study of the basis of the retinal thickening and of the SMM-189 modulation of it is needed.

#### 2.2.4. Depression and Fear

**Figure 10:**
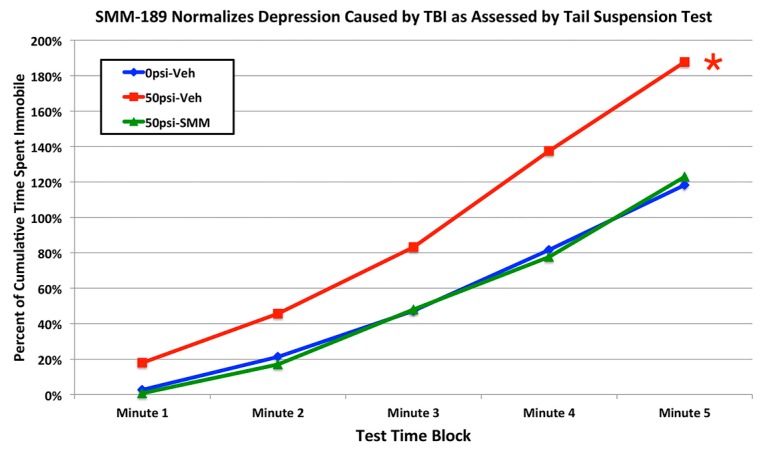
Graph showing the effects of 50-psi blasts compared to 0-psi blasts in vehicle-treated mice on the tail suspension test of depression six to eight weeks after blast. Data are presented as cumulative immobility per consecutive one-minute block. Note that immobility, reflecting a depression-like behavior, is increased in 50-psi mice compared to 0-psi mice. By contrast, depression is normalized in 50-psi mice treated with SMM-189. The asterisk indicates a significant difference between vehicle-treated 50-psi and sham mice.

We have previously shown that mild TBI in mice produced by our approach yields increased depression when assessed one-two months after blast by the tail suspension test [15]. This was also true in a cohort of vehicle-treated mice with 50-psi blast compared to vehicle-treated mice with sham blast (Figure 10), in which depression was significantly greater in vehicle-treated 50-psi mice than in vehicle-treated sham blast mice (*p* = 0.012). SMM-189 treatment alleviated the depression (immobility) observed in 50-psi mice, normalizing it to a level that was statistically indistinguishable from that in the sham-blasted vehicle-treated mice (*p* = 0.951), and significantly less than in the vehicle-treated 50-psi mice (*p* = 0.011).

**Figure 11:**
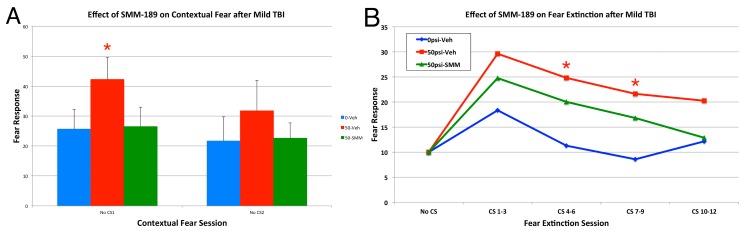
Graphs showing the effects of 50-psi blasts compared to 0-psi blasts in vehicle-treated mice compared to 50-psi mice treated with SMM189 on contextual fear (**A**) and fear extinction (**B**) subsequent to acquisition of conditioned fear to an auditory cue signaling impending shock, six to eight weeks after blast. Freezing during successive 30-s time blocks over the first 60 s of the first contextual fear test (**A**) is increased overall in the vehicle-treated 50-psi mice compared to vehicle-treated 0-psi mice, and the difference for the first 30 s block is significant (red asterisk). Note that contextual fear is normalized in 50-psi mice treated with SMM189. Similarly, fear responses to the CS are elevated in vehicle-treated 50-psi mice compared to vehicle-treated 0-psi mice (**B**) over the three days of fear extinction testing. Note that 50-psi mice do not differ from 0 to 30 psi mice in fear acquisition, but do show more fear retention and less fear extinction [15]. Fear responding during extinction is attenuated in 50-psi mice treated with SMM189, and declines to control levels by the CS presentations 9–12. Fear extinction performance has been standardized to show CS responses above contextual responses, and each consecutive three-trial block has been averaged over the 12 CS presentations.

**Figure 12:**
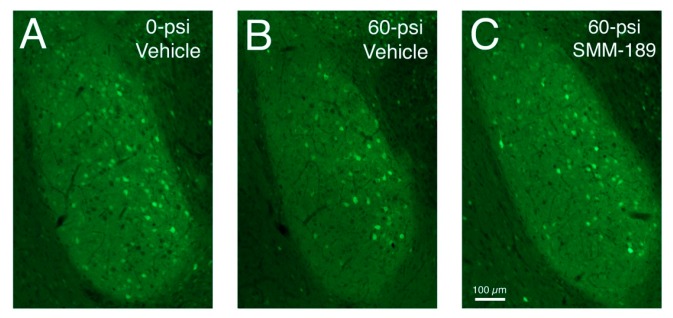
Images of sections through the basolateral amygdala on the blasted side from mice engineered to express enhanced yellow fluorescent protein (EYFP) in Thy1-enriched telencephalic neurons. Note that the basolateral amygdala (BLA) in the vehicle-treated 0-psi mouse (**A**) contains numerous Thy1-EYFP+ neurons, while the BLA in the vehicle-treated 50-psi mouse (**B**) contains fewer. Thy1+ neurons in BLA are increased in SMM-189-treated 50-psi mice (**C**) above that seen in the vehicle-treated 50-psi mouse.

We have also previously shown that three to eight weeks after 50–60 psi left cranial blast, mice show increased contextual fear, and heightened retention of learned fear [15]. In the present study, we found that SMM-189 treatment diminished both forms of learned fear (Figure 11). For example, vehicle-treated 50-psi mice showed a significant increase in learned contextual fear compared to vehicle-treated sham mice when re-introduced into their training context over the first minute of the first fear extinction session (*p* = 0.012). SMM-189 treatment reduced this significant increase in contextual fear, so that it was statistically indistinguishable from that shown by sham-blasted mice receiving vehicle (*p* = 0.904), and significantly less than that shown by the vehicle-treated 50-psi mice (*p* = 0.018). Secondly, vehicle-treated 50-psi mice also showed a large and significant overall increase across trials in learned fear responses to the conditioned stimulus (CS) during the three extinction sessions compared to vehicle-treated sham mice (*p* = 0.000147), with significant specific elevations evident during CS presentations 4–6 (*p* = 0.023) and 7–9 (*p* = 0.028). Although responses to the CS in the SMM-189-treated mice remained greater than in the vehicle-treated sham mice across trials overall (*p* = 0.049), they reached sham levels by the 10th–12th CS presentations, and were not significantly greater than in sham for any of the four grouped CS presentations. Moreover, they trended overall toward being significantly different than in the 50-psi vehicle treated mice (*p* = 0.051). The morphological basis of the SMM-189 benefit is uncertain, but we have previously noted that the Thy1+ fear-suppressing neurons in the basolateral amygdala are reduced by about 25% in their abundance by 50–60 psi blasts [15]. In the present study, we confirmed a 20% reduction in Thy1+ neurons in BLA in six vehicle-treated mice two months after 60-psi blast. SMM-189 treatment in ten 60-psi blast mice rescued this loss of Thy1+ fear suppressing neurons in the basolateral amygdala, so that their abundance in BLA was indistinguishable from that seen in vehicle-treated mice receiving 0–30 psi blasts (Figure 12).

## 3. Discussion

The axonal pathology we observed after 50–60 psi blasts in our model is accompanied by microglial activation in the damaged tracts and in their associated terminal fields over the first week after blast [15,16]. The observed microglial activation is likely to occur via their Toll-like 4 receptors in response to small soluble factors (e.g., glutamate, ATP) and/or molecules (e.g., cytoskeletal elements, proteolytic breakdown products of cytoskeletal and other cellular elements, and lipids) released by or associated with damaged axons, their terminals, and their ensheathing myelin [43,44]. Many authors have suggested that persistent microglial activation initiates an inflammatory cascade that worsens the outcome after TBI [7,11,14]. Because of the evidence for this [7,8,9,10,11,12,13,14] and the evidence that microglia can be modulated by their CB2 receptors [18,22], we tested the ability of the novel CB2 receptor inverse agonist SMM-189 to diminish the deficits we saw with 50–60 psi blasts. Our *in vitro* analysis showed that SMM-189 converted microglia from a pro-inflammatory M1 phenotype to a pro-healing M2 phenotype, and reduced cytokine and chemokine expression characteristic of LPS-activated pro-inflammatory microglia. We further found that daily administration of SMM-189 for two weeks beginning shortly after blast greatly improved the motor, visual, and emotional outcomes after 50-psi blasts when tested at three to eight weeks after blast, and repaired at least some of the underlying brain injury possibly contributing to these deficits. The SMM-189 benefit was associated with CREB activation in microglia, which would be caused by its inverse agonist action at microglial CB2 receptors and the resulting activation of signaling pathways leading to cAMP phosphorylation. Moreover, nuclear translocation of pCREB is thought to drive M1 activated microglia to the M2 phenotype and thereby underlie both an anti-inflammatory effect and a pro-repair effect of CB2 inverse agonists [35,36,38]. Our results thus suggest consideration and further testing of CB2 inverse agonists as a novel therapeutic approach for treating mild TBI.

### 3.1. Motor Deficits

The deficits with our focal blast model of mild TBI appear to stem, in large part, from axon injury, since overall neuron loss with our model appears to be minimal [15]. For example, rotarod and open field deficits may all relate to damage to descending tracts. Of these, we examined only the corticospinal tract, for which the damage appeared to be remedied by SMM-189 treatment. Deficits in other circuits are likely to have contributed to these motor deficits as well. For example, vestibular or cerebellar function can contribute to performance on rotarod, and extrapyramidal (e.g., basal ganglia) and/or motivational circuitry (e.g., nucleus accumbens) are likely to influence motor behavior in an open field arena. If so, it would be of interest to know if SMM-189 has a beneficial effect on these brain regions as well.

### 3.2. Visual Deficits

Various animal models have shown that optic nerve damage, ganglion cell loss and retinal thinning can result from TBI itself, presumably due to the effect of the concussive forces acting on the optic nerve and eye [45,46,47,48,49]. Similarly, TBI even without concurrent ocular trauma commonly causes optic nerve and retinal injury, and visual deficits in humans as well [50,51,52]. The decreased visual acuity and contrast sensitivity in the left eye after left cranial blasts in our model too are very likely to result, at least in part, from injury to axons in the left optic nerve and right optic tract, as well as possibly directly to the retina itself from the cranial transit of the pressure wave [16]. Interestingly, the loss of contrast sensitivity was greater than the acuity deficit for the left eye. Acuity and contrast sensitivity reflect different aspects of visual functioning, and contrast sensitivity deficits at low spatial frequency have been reported without comparable deficits in spatial acuity measured at high contrast, for example in humans with aging, diabetes or glaucoma [53,54,55]. Moreover, contrast sensitivity loss without commensurate high contrast spatial acuity loss is often an early sign of optic nerve injury and ganglion cell loss [56,57,58], although this has not been specifically studied after TBI. In any event, SMM-189 significantly attenuated the deficit in contrast sensitivity for the left eye, and appeared to reduce the acuity deficit as well. This benefit was associated with normalization of thickness of all retinal layers that had been adversely affected by 50-psi left-side cranial blast. It, thus, seems likely that the SMM-189 rescue of left eye deficits is mediated, at least in part, by its beneficial effect on the left retina. Some of this benefit may have been mediated by mitigation of the left optic nerve injury we have observed in our model [16]. Further studies will be needed to evaluate the contributions of these different factors, including ganglion cell loss, to injury with 50-psi blast and its amelioration with SMM-189. Although the blasts were directed to the left hemisphere, injury to visual areas of the right side of the brain, which receive visual information largely from the left eye in mice [59], may have also occurred by contrecoup injury. Thus, we cannot rule out a role of central injury in the left eye deficits, and rescue of visual areas of the right side of the brain may at least be part of the basis of the SMM-189 benefit for left eye-mediated visual function.

The basis of the visual deficits in the right eye is less certain. In mice, about 90% of optic nerve axons cross to the contralateral side of the brain and only 10% are uncrossed [59]. Blasts delivered to the left hemisphere may have thus injured visual areas of the left side of the brain critical to right eye visual performance. We also found thickening of the right inner retina, which may have contributed to the visual deficit. One possible explanation for the right eye retinal thickening is edema. Displacement of the head rightwards by the blast may have pushed the right eye into the foam [15], thereby producing ocular trauma, which has been shown to yield retinal edema in humans in many cases of mild closed-globe trauma [60]. We, however, did not notice any obvious corneal edema, which typically accompanies blunt ocular trauma in humans and mouse models [60,61]. This does not, however, preclude the possibility of retinal edema in the right eyes of our mice. In any event, SMM-189 slightly diminished the inner retinal thickening so that it was no longer significantly different than in sham eyes. This may have contributed to the rescue of right eye acuity and contrast sensitivity seen after 50-psi blast. Nonetheless, mitigation of injury to brain visual areas by SMM-189 may also be a contributor to the functional rescue for the right eye, given the only small improvement in retinal thickness with SMM-189 and the large benefit for vision. In this regard, it should be noted that the retinal edema may have been more severe and the SMM-189 thickness benefit greater at the four week post blast time point that visual function was measured, than at the 11 week post blast time point at which retinal measurements were performed. Further studies will be needed to elucidate the basis of the visual deficits in the right eye with 50-psi blast and their amelioration with SMM-189. It will also be important to determine why blast to the left side of the cranium in our model yields a greater contrast sensitivity deficit in the left eye and a greater acuity deficit in the right eye.

### 3.3. Fear and Depression

The heightened fear retention exhibited by the mice in our model is likely to be attributable to the reduction in Thy1+ neurons in the basolateral amygdala, as we previously have reported [15]. Activity in the Thy1+ neurons of BLA during fear acquisition reduces fear memory, and activation of these neurons during presentation of learned fear stimuli enhances extinction of fear responses to the fear stimulus [62]. Reduced activity in these neurons would thus be expected to yield the outcomes we see after mild TBI in mice—no effect on fear acquisition but subsequent heightened responding during CS-alone presentations and reduced extinction of fear responding to CS alone. Whether the reduced number of Thy1+ BLA neurons in our EYFP reporter mice reflects true loss of these neurons or a dysfunction manifesting as diminished Thy1 expression is uncertain. Moreover, it is uncertain if TBI injures the Thy1+ BLA neurons directly, or whether the abnormality in Thy1+ BLA neurons reflects TBI injury to the medial prefrontal cortex neurons that preferentially innervate them [63]. More information is needed about the role of the Thy1+ neurons of BLA and their input from medial prefrontal cortex in the learned fear enhancement we observed after mild TBI, and the means by which SMM-189 reduces this deficit. Similarly, further studies are needed to elucidate the nature of the brain injury underlying the depression that occurs after 50-60 psi blasts, and its amelioration by SMM-189.

### 3.4. Summary and Conclusions

We found that daily administration of SMM-189 for two weeks beginning shortly after a mild TBI event greatly reduced motor, visual, and emotional deficits, and repaired at least some of the underlying brain injury possibly contributing to these deficits. Our results thus suggest consideration and further testing of CB2 inverse agonists as a novel therapeutic approach for treating deficits after mild TBI. Importantly, dose and timing need investigation so as to optimize the outcome and determine the critical window in which treatment is most effective. The extent of benefit also needs to be examined for additional motor, sensory and neuropsychiatric endpoints, as well as evaluated in other models of TBI. Finally, it would be valuable to determine the morphological basis of the different deficits and their rescue with SMM-189. For example, it would be useful to know if the SMM-189 benefit for left eye vision involves rescue of optic nerve axons and whether a pro-healing M2 microglial phenotype is critical to this axonal rescue.

## 4. Experimental Section

### 4.1. Studies of Human Microglia in Vitro

The ability of the CB2 receptor inverse agonist SMM-189 to have an anti-inflammatory effect on human microglia *in vitro* was evaluated in two types of studies. First, we examined the effect of SMM-189 on the production of cytokines and chemokines by primary human microglia activated by the M1 stimulant lipopolysaccharide (LPS). Secondly, we examined the effect of SMM-189 on the expression of M1* versus* M2 microglial markers by immortalized human microglia exposed to either the M1 pro-inflammatory stimulant LPS or the M2 anti-inflammatory stimulant interleukin-4 (IL-4).

#### 4.1.1. Cytokine and Chemokine Production by Primary Human Microglia

Primary human microglia (Clonexpress #HMG 030), maintained according to supplier recommendations, were seeded on 96-well polystyrene culture plates at a density of 300,000 cells/mL (100 µL per well) and incubated at 37 °C in 5% CO_2_–95% air for 24 h to allow cell attachment. SMM-189 was prepared in dimethyl sulfoxide (DMSO) at 100× concentration and mixed 1:100 in media containing 1% FBS (fetal bovine serum). Culture plates were removed from the incubator and the complete growth medium was replaced with 50 µL medium containing 1% FBS and LPS at 1 µg/mL, or without LPS in the case of control wells. They were then returned to the incubator for one hour, removed, and 50 µL of medium containing vehicle alone or sufficient SMM-189 to yield a 9.8 µM concentration in the case of SMM-189 (the EC_50_ for the primary human microglia) was added to pre-determined wells. After 18 hours in the incubator, media supernatants were assayed for cytokines and chemokines using the Human ProInflammatory 9-Plex Tissue Culture Kit (#K15007B-1, Meso Scale Discovery, Rockville, MD, USA,) and the Human Chemokine 9-PlexTissue Culture Kit (Meso Scale Discovery, #K15001B-1), using the SECTOR Imager 2400 (Meso Scale Discovery), according to the manufacturer’s instructions. Measurements were made in quadruplicate. In three instances out of 20 conditions, single missing values were mean filled for the condition to balance statistical analysis (IFN-γ for LPS plus SMM-189, IL-8 for control, and MCP-1 for control).

#### 4.1.2. M1* versus* M2 Marker Expression by Immortalized Human Microglia

Immortalized SV40 human microglia (Applied Biological Materials Inc., Richmond, BC, Canada) were maintained according to supplier recommendations. Microglia were seeded on 96-well Meso Scale Discovery high bind plates (L15XB-3) at a density of 400,000 cells/mL (20,000 cells in 50 µL per well) and incubated at 37 °C in 5% CO_2_/95% air overnight to allow cell attachment. The following morning, plating medium was carefully removed and replaced with 100 uL of medium containing 1% FBS. After 24 h, LPS (L2630, Sigma-Aldrich, St. Louis, MO, USA) was added to a final concentration of 100 ng/mL in some wells, while others received concurrent LPS and SMM-189 at a previously determined EC_50_ (13.4 µM). Twenty-four hours later, expression of M1 markers (CD11b, CD45, CD80; #ab64347, ab1176, ab8239, Abcam, Cambridge, MA, USA) was assessed using 30 µL per well of antibody in PBS at a concentration of 2 µg/mL. After a two-hour incubation on a shaker plate at 130 rpm, wells were gently washed using 150 µL PBS, and 30 µL per well of secondary antibody containing SULFOTAG in PBS was added at a concentration of 2 µg/mL. Plates were then incubated for another two hours, washed gently three times with 150 µL per well of PBS, and read in 2X surfactant-free read-buffer using a SECTOR Imager 2400 (Meso Scale Discovery). In a second set of studies, expression of M2 markers (CD206 and CD209; ab8918, ab59192, Abcam) was assessed after treatment of SV40 human microglia at the same cell plating density with human recombinant IL-4 (SRP3093, Sigma-Aldrich) at 20 ng/mL alone or in combination with SMM-189 (13.4 µM). The overall experimental sequence for assessment of M2 markers was as for M1 markers after LPS stimulation. Finally, the effect of different M1 or M2 microglial stimulants on cell surface CB2 on the immortalized human microglia was assessed using an anti-CB2 antibody (ab561, Abcam), with microglia again plated at 20,000 cells in 100 µL per well. The overall experimental sequence for assessment of CB2 was the same as used for M1 or M2 markers. The microglial stimulants used were: human recombinant interferon-gamma (IFN-γ) at 20 ng/mL (285-IF-100, R&D Systems, Minneapolis, MN, USA), recombinant human interleukin-10 (IL-10) at 20 ng/mL (217-IL-005, R&D Systems), recombinant human interleukin-1-beta (IL-1β) at 50 ng/mL (I9401, Sigma-Aldrich), LPS at 100 ng/mL, and recombinant human IL-4 20 ng/mL. Measurements were made in triplicate. Data were normalized to percentage expression compared to control wells (denoted as M0).

### 4.2. Studies of Mild TBI in Mouse

#### 4.2.1. Animals

Three-month old male mice were subjected to TBI caused by blast intensities ranging from 0 to 50 psi above atmospheric pressure (*i.e*., 14-psi), and the outcome evaluated behaviorally and histologically. Adult male C57BL/6 mice were used to study the behavioral and morphological effects of mild TBI alone or in combination with SMM-189 treatment. SMM-189 was formulated in a vehicle containing ethanol:Cremophor:0.9% saline (5:5:90), which are categorized as “Generally Regarded As Safe” (GRAS) excipients by the Food and Drug Administration (FDA). The C57BL/6 mice were either purchased from JAX (Bar Harbor, ME, USA), and/or taken from a colony maintained from C57BL/6 founders from JAX. We also used mice conditionally expressing enhanced yellow fluorescent protein (EYFP) in Thy1-expressing telencephalic neurons of the *emx1* lineage to histologically evaluate the effects of blast alone or in combination with SMM-189 treatment. As described previously, in the progeny of a cross between a floxed Thy1-EYFP reporter mouse and an *emx1*-Cre driver mouse, nearly all excitatory cortical neurons, many amygdalar neurons, and 5% of retinal ganglion cells express EYFP [15,64,65]. The EYFP reporter mice and the *emx1*-Cre driver mice were purchased from the Mutant Mouse Regional Resource Centers (MMRRC) or JAX, respectively, and colonies were established and maintained at the University of Tennessee Health Science Center (UTHSC). All animal studies were performed in accordance with UTHSC Institutional Animal Care and Use Committee protocol #13-156.0 (approved: January 2011–Present), and complied with the National Institutes of Health and Society for Neuroscience guidelines.

In treatment studies, we injected mice with 6 mg/kg SMM-189 i.p. or vehicle daily for 14 days beginning two hours after 50-psi blast. The dose used was chosen based on studies of uptake in rodent brain of structurally similar tri-aryl CB2 compounds [40]. From those studies, we estimated a 4.6 µM SMM-189 concentration in mouse brain within hours of injection of a 6 mg/kg dose, which would be more than adequate for CB2 receptor activation given its 2.9 nM affinity [39].

#### 4.2.2. TBI Methods

The over-pressure air blast used to create mild TBI was delivered by a small horizontally mounted air cannon system [15,16,61], which utilized a modified paintball gun (Invert Mini, Empire Paintball, Sewell, NJ, USA). Anesthetized mice were secured within a foam rubber sleeve inside a pair of Plexiglas tubes, with the targeted head region positioned in the center of a 7.5 mm diameter hole in the outer tube, 4–5 mm from the blast cannon barrel opening. The part of the mouse exposed to the blast was restricted to a 7.5 mm diameter area on left side of the mouse cranium between ear and eye, as described previously [15,16]. The rest of the mouse was completely shielded from the blast by a Plexiglas mouse holder, and a foam rubber sleeve surrounding the mouse cushioned the non-blast side of the mouse. The system is designed so that the blast pressure experienced by the mouse head can be set to a defined value. Blasts of 20-, 40- and 60-psi, for example, have times to peak of 2.5, 4.4 and 5.0 milliseconds (ms), respectively, with total blast duration being 5.5, 10.5, and 15.0 ms, respectively.

#### 4.2.3. Behavioral Studies-Motor Assessment

Rotarod analysis was carried out using a San Diego Instruments (San Diego, CA, USA) rodent rotarod. For the rotarod task, revolutions per minute (RPM) increased from 0 to 30 over a four-minute period, and 30 RPM was then maintained for another two minutes. The first rotarod session was a two-trial training session two days before blast, followed by a two-trial test session the following pre-blast day. Mice were then tested again the day after blast, one week after blast, and two weeks after blast. Time to fall was the measure of rotarod performance. We also conducted automated 30-min assessment of open field behavior, using a Noldus EthoVision video tracking system to record and digitize the mouse movements (Noldus Information Technology, Wageningen, The Netherlands), and the SEE software of Drai and Golani [66] to analyze the mouse motor behavior, as described previously [66,67,68,69,70,71]. Open field sessions were conducted the day before, the day after, one week after, and two weeks after blast.

#### 4.2.4. Behavioral Studies-Visual Acuity and Contrast Sensitivity

We used a virtual reality Optomotry System (CerebralMechanics Inc., Lethbridge, AB, Canada) to measure acuity and contrast sensitivity [72]. Spatial frequency thresholds were measured by systematically increasing the spatial frequency of the grating at 100% contrast until animals no longer displayed tracking behavior. Contrast sensitivity was determined at 0.042 cycles/degree spatial frequency. Drum rotation was random from trial to trial, and the experimenter determined from the video monitor if the animal moved clockwise or counterclockwise for a given trial. Stimulus presentations were computer-controlled and the experimenter remained blinded to the stimuli seen by the animal. As the optokinetic response is driven by temporal to nasal stimulus movement, clockwise movement of the pattern tests the left eye, and counterclockwise movement tests the right eye.

#### 4.2.5. Tail Suspension Depression Test

Depression is a prominent symptom after TBI in humans [73]. To assess depression-like behavior in mice, the tail suspension test was used [15,74]. Mice suspended by their tail eventually stop attempting to escape and become immobile, with a depressive-like state indicated by a short latency to immobility and longer durations of immobility over the test period. For this test, each mouse was suspended above a solid surface by the use of vinyl adhesive tape applied to the tail so that its body dangled in the air, facing downward. Immobility was recorded and analyzed over a 5 min period using a digital video camera interfaced with a computer and automated software (FreezeFrame, Coulbourn, Whitehall, PA, USA).

#### 4.2.6. Fear Acquisition and Extinction Tests

Human victims of mild TBI tend to show perseverative fear memories [75]. We tested for increased fear retention in our mild TBI mice following fear conditioning using a Pavlovian paradigm [15]. The fear-conditioning chamber possesses clear polycarbonate walls and a stainless steel grid floor (MED Associates, Model ENV-008), and is fitted with a video camera interfaced with a computer. Foot shock stimuli and conditioned freezing responses were controlled, collected, and analyzed using automated software (FreezeFrame, Coulbourn, Whitehall, PA, USA). Five minutes after being placed in the training chamber, mice received five fear training trials, each consisting of a 30-s tone (12 kH) conditioned stimulus (CS) co-terminating with a 0.250-s, 0.4-mA foot shock (unconditioned stimulus), with an intertrial interval of two minutes. On each of the following three days, mice were given extinction sessions, each consisting of tone-alone test trials, with freezing again the measure of fear, at an intertrial interval of two minutes. The CS presentations were preceded by a three-minute assessment of responding to the fear context.

### 4.3. Histological Studies

#### 4.3.1. Tissue Fixation

Histological analysis was carried out on fixed tissue to determine the effects of mild TBI alone or with SMM-189 treatment on brain, spinal cord, and retina using a number of approaches: (1) immunolabeling of free-floating brain sections or slide-mounted cryostat sections of spinal cord or eye for IBA1 (ionized calcium-binding adapter molecule-1), major histocompatibility complex (MHC) class II antigen, phosphorylated-cyclic AMP response element binding protein (pCREB), and protein kinase C-gamma (PKC-γ); (2) toluidine blue staining of one-micron sections through retina; (3) phenylenediamine (PPD) staining of one-micron sections through the optic nerve; and (4) EYFP visualization in the EYFP reporter mice. Mice were deeply anesthetized (avertin; 0.2 mL/10 g body weight), the chest opened, and 0.1 mL of heparinized saline (800 U.S.P. units/mL) injected into the heart. They were then perfused transcardially with 40 mL of 0.9% NaCl in 0.1 M sodium phosphate buffer at pH 7.4 (PB), followed by 200 mL of 4% paraformaldehyde, 0.1 M lysine–0.1 M sodium periodate in 0.1 M PB at pH 7.4 (PLP). The brains were removed and stored for at least 24 h in a 20% sucrose/10% glycerol solution at 4 °C, and sectioned frozen on a sliding microtome in the transverse plane at 35 µm. Each brain was collected as 12 separate series in 0.1 M PB with 0.02% sodium azide and stored until processed for immunohistochemistry. A one in six series of brain sections from each mouse was mounted as sectioned, and subsequently stained for cresyl violet. After perfusion and brain removal, the eyes and optic nerves were removed. Optic nerves were in some cases transferred to 4% paraformaldehyde–0.5% glutaraldehyde in 0.1 M PB, and then stored at 4 °C until embedded in plastic, sectioned at 1 µm, and stained with PPD. For eyes, corneas were then dissected free, the lens removed, and eyecups fixed further in either paraformaldehyde–0.5% glutaraldehyde in 0.1 M PB overnight or PLP for two hours. Eyes fixed with 4% paraformaldehyde–0.5% glutaraldehyde in 0.1 M PB were washed, dehydrated, and embedded in plastic, sectioned at 1 µm, and stained with toluidine blue. Eyes fixed with PLP (with optic nerve attached in some cases) were washed, stored at 4 °C in 20% sucrose in 0.1 M PB until sectioned at 30 µm using a cryostat. After brain/eye removal, spinal cords were exposed by ventral laminectomy and postfixed overnight in PLP at 4 °C. Thoracic cord was then dissected free and stored at 4 °C in 20% sucrose in 0.1 M PB until sectioned at 30 µm using a cryostat. On-the-slide immunolabeling for eyes and cords was as described previously [76].

#### 4.3.2. Immunohistochemical Studies

We used peroxidase-antiperoxidase (PAP) single labeling, and single and multiple label immunofluorescence, as described previously [77]. We used immunolabeling with an antibody selective for the microglial marker IBA1 to determine whether microglia showed morphological alterations reflective of activation [78]. Additionally, we used immunolabeling with antibodies for MHC Class II antigen to evaluate microglial activation, since it is particularly elevated in M1-activated microglia [79,80]. IBA1 was detected with a Wako rabbit polyclonal antibody against the conserved IBA1 *C*-terminus, and the MHC Class II antigen was detected with the mouse monoclonal OX6 antibody from BD Pharmingen [81]. To detect corticospinal tract fibers in the spinal cord, we performed immunolabeling for PKC-γ using a rabbit polyclonal antibody from Abcam [82,83,84]. Finally, CB2 receptor inverse agonists such as SMM-189 boost cAMP levels, and as a result increase activated phosphorylated-CREB (pCREB) [38]. We used immunolabeling for pCREB (Rabbit Anti-Phospho-Ser133 CREB; PhosphoSolutions, Aurora, CO, USA, Catalog Number: p1010-133) to assess increases in nuclear activated CREB in activated microglia following SMM-189 treatment in mice subjected to 50–60 psi blasts.

For PAP immunolabeling, sections were incubated for 24 h at room temperature in primary antibody diluted 1:2000–1:5000 with 0.8% Triton X-100/0.01% sodium azide/0.1 M PB (PBX). Sections were then rinsed and incubated in donkey anti-mouse IgG or anti-rabbit IgG (depending on the primary antibody) diluted 1:200 in PBX, followed by incubation in the appropriate mouse or rabbit PAP complex diluted 1:1000 in PBX, with each incubation at room temperature for one hour. The sections were rinsed between secondary and PAP incubations in three five-minute washes of PB. Subsequent to the PAP incubation, the sections were rinsed with three to six 10-min washes in 0.1 M PB, and a peroxidase reaction using diaminobenzidine tetrahydrochloride (DAB) was carried out. Sections were incubated in 5 mL of 0.05 M imidazole/0.05 M cacodylate buffer (pH 7.2) containing 5 mg DAB for 10 min and then incubated for an additional 10 min after adding 20 µL of 3% hydrogen peroxide. Sections were then rinsed in distilled water, transferred to 0.1 M PB, mounted onto gelatin-coated slides, dried, dehydrated, and coverslipped with Permount^®^. Immunofluorescence was carried out to detect multiple fluorescent markers, typically IBA1, OX6 or pCREB in optic tract or nerve in the *emx1-*Thy1-EYFP reporter mice. Slide-mounted cryostat sections of eye and optic nerve or free-floating sections of brain were incubated overnight in primary antibody at room temperature, as described previously [76,85,86]. The sections were then rinsed three times, and incubated for two hours at room temperature (with gentle agitation in the case of free floating) in an Alexa 594-conjugated goat anti-mouse or rabbit IgG (Molecular Probes, Eugene, OR, USA). Sections were rinsed three times in 0.1 M PB afterwards, free-floating sections were mounted on gelatin-coated slides, and all slides were coverslipped with ProLong^®^ antifade medium (Molecular Probes, Eugene, OR, USA). Sections were viewed and images captured using a Zeiss 710 confocal laser-scanning microscope (CLSM) (Jena, Germany).

#### 4.3.3. Thy1-EYFP–*Emx1*-Cre Reporter Mice

*Thy1*-EYFP–*emx1*-cre mice were transcardially perfused with fixative and sectioned as described above. Sections were mounted onto gelatin-coated slides, dried, dehydrated, and coverslipped with ProLong^®^ antifade medium (Molecular Probes, Eugene, OR, USA). Sections were viewed and images captured using a Zeiss 710 CLSM. These mice were used to assess injury to EYFP+ fiber tracts, and EYFP+ cortical and amygdaloid neurons. Immunolabeling for GFP (Mouse Anti-GFP Monoclonal Antibody; Rockland Immunochemicals, Gilbertsville, PA, USA, catalog number: 600-301-2159) using the PAP method was used to render EYFP detectible by transmitted light microscopy in some cases.

#### 4.3.4. Morphometry

To measure retinal thickness, images of retinal cross sections were taken from at least three separate fields of the central retina. These images were then measured blinded for total retinal thickness, inner nuclear layer (INL) thickness, and inner plexiform layer (IPL) to nerve fiber layer (NFL) thickness. Total retinal thickness was measured from the outer edge of the retinal pigmented epithelium (RPE) to the inner edge of the NFL. Inner plexiform layer to NFL measurements were taken from the inner edge of the INL to the vitread edge of the NFL. The resulting measurements from each eye were averaged to yield a thickness for each retina, as well as for its sublayers. For quantification of the dorsal corticospinal tract (dCST), images of transverse sections were captured of the entire section or the dorsal funiculus at upper-mid thoracic levels from PKC-gamma immunostained tissue. Blinded image analysis using National Institutes of Health Image J (Bethesda, MD, USA) was then used to threshold the labeled fibers in the left and right dCST, and assess the relative fiber abundance in the selected area. Finally, images of the basolateral amygdala (BLA) in the Thy1+ EYFP-reporter mice were captured using a Zeiss 710 confocal laser-scanning microscope (CLSM). Blinded counts of Thy1-EYFP+ neurons and BLA area measurements were made using the particle counting and area measurement functions in Image J.

### 4.4. Statistical Analysis

ANOVA followed by planned comparisons using *post-hoc* Fisher PLSD (Protected Least Significant Difference) tests was used to analyze human primary microglia data, immortalized human microglia M1 and M2 data, mouse behavioral data, and mouse histological data. Overall ANOVA were significant for the majority of tests, except in cases in which the 50-psi deficit in vehicle-treated mice was relatively small and/or in which rescue with SMM-189 was substantial. These include the motor endpoints (rotarod, maximum speed and turn radius), left retina thickness, left inner retina thickness, and all right retina thickness measurements. CB2 receptor data for immortalized human microglia were analyzed using *t*-tests. Results are presented as group mean ± SEM.

## 5. Conclusions

In summary, motor, visual, and emotional deficits are common outcomes in humans after mild TBI, and treatment options that mitigate these deficits are limited. We have found that SMM-189 delivered daily in the two-week period after TBI greatly reduces these deficits in a mouse model of mild TBI. Our evidence suggests that SMM-189 produces a prominent benefit after mild TBI, apparently by converting M1-activated microglia to the M2 phenotype. Whether the M2 conversion is beneficial because of its anti-inflammatory effect and/or its reparative effect requires further study [35,36,38], as does a more detailed time course of the axonal and neuronal rescue associated with SMM-189 treatment. In any case, our findings recommend further evaluation of CB2 inverse agonists as a novel therapeutic approach for treating mild TBI.
